# Priming the Abscopal Effect Using Multifunctional Smart Radiotherapy Biomaterials Loaded with Immunoadjuvants

**DOI:** 10.3389/fonc.2018.00056

**Published:** 2018-03-12

**Authors:** Michele Moreau, Sayeda Yasmin-Karim, Sijumon Kunjachan, Neeharika Sinha, Felix Gremse, Rajiv Kumar, Kwok Fan Chow, Wilfred Ngwa

**Affiliations:** ^1^Department of Radiation Oncology, Dana–Farber Cancer Institute, Boston, MA, United States; ^2^Department of Physics and Applied Physics, University of Massachusetts Lowell, Lowell, MA, United States; ^3^Department of Radiation Oncology, Brigham and Women’s Hospital, Boston, MA, United States; ^4^Institute for Experimental Molecular Imaging, RWTH Aachen University, Aachen, Germany; ^5^Electronic Materials Research Institute, Northeastern University, Boston, MA, United States

**Keywords:** radiotherapy, immunotherapy, biomaterials, abscopal effect, antigen-presenting cell

## Abstract

In this study, we investigate the use of multifunctional smart radiotherapy biomaterials (SRBs) loaded with immunoadjuvants for boosting the abscopal effect of local radiotherapy (RT). SRBs were designed similar to currently used inert RT biomaterials, incorporating a biodegradable polymer with reservoir for loading payloads of the immunoadjuvant anti-CD40 monoclonal antibody. Lung (LLC1) tumors were generated both on the right and left flank of each mouse, with the left tumor representing metastasis. The mice were randomized and divided into eight cohorts with four cohorts receiving image-guided RT (IGRT) at 5 Gy and another similar four cohorts at 0 Gy. IGRT and Computed Tomography (CT) imaging were performed using a small animal radiation research platform (SARRP). Tumor volume measurements for both flank tumors and animal survival was assessed over 25 weeks. Tumor volume measurements showed significantly enhanced inhibition in growth for the right flank tumors of mice in the cohort treated with SRBs loaded with CD40 mAbs and IGRT. Results also suggest that the use of polymeric SRBs with CD40 mAbs without RT could generate an immune response, consistent with previous studies showing such response when using anti-CD40. Overall, 60% of mice treated with SRBs showed complete tumor regression during the observation period, compared to 10% for cohorts administered with anti-CD40 mAbs, but no SRB. Complete tumor regression was not observed in any other cohorts. The findings justify more studies varying RT doses and quantifying the immune-cell populations involved when using SRBs. Such SRBs could be developed to replace currently used RT biomaterials, allowing not only for geometric accuracy during RT, but also for extending RT to the treatment of metastatic lesions.

## Introduction

Radiotherapy (RT) is employed in the treatment of over 50% of cancer patients (1). Despite continuing developments to increase therapeutic efficacy, RT is still significantly limited by normal tissue toxicity and is mainly utilized for treating localized disease sites (2–4). On the other hand, immunotherapy uses immune-modulating pharmaceutical agents to stimulate either an innate or adaptive immune response to kill cancer cells, including cancer metastasis (5, 6). However, normal tissue toxicities or immune-related adverse events are also a limitation to the use of immunotherapies (6–8). More recently, there are a growing number of studies combining RT with immunotherapy to enhance both local and metastatic tumor cell kill leveraging the immune-mediated abscopal effect (9–13). This abscopal effect can enable the killing of metastatic cells, distant from the irradiated site (12). However, clinical trials show limited treatment benefit for lung tumors with abscopal response rates remaining low due to immunosuppression (4, 13). In previous work (3), the use of smart RT biomaterials (SRBs) loaded with immunoadjuvants was proposed to boost abscopal response rates, with the potential to benefit many more patients, especially those with metastatic disease (2).

The rationale for using SRBs is that they can sustainably deliver immunoadjuvant payloads directly into the tumor subvolume. The sustained delivery enhances the potential to overcome immunosuppression, especially given the persistent and contemporaneous presence of antigen and adjuvant signaling in the tumor microenvironment, during RT (13–15). The *in situ* delivery of payloads from the SRBs will allow direct delivery of the immunoadjuvant payload into the tumor microenvironment, with the potential to significantly minimize systemic or overlapping toxicities (16), which are currently a critical barrier for other approaches (17, 18). Furthermore, the SRBs could be designed to simply replace currently used inert RT biomaterials (e.g., spacers, fiducials, etc.) to serve as multifunctional SRBs. These SRBs can help ensure geometric accuracy during treatment, but also deliver immunoadjuvants to boost the abscopal response (18, 19), all at no additional inconvenience to cancer patients (2, 8, 13).

The goal of this study is to investigate the use of SRBs loaded with immunoadjuvants in boosting abscopal response rates for lung cancer. Our results demonstrate for the first time, the potential of SRBs loaded with CD40 mAbs in significantly boosting the abscopal and survival in animal models of lung cancer.

## Materials and Methods

### Materials

Poly (lactic-co-glycolic) acid (PLGA) (M.W.:50–50 kD_a_), dimethyl sulfoxide (DMSO), chloroform anhydrous, and fluorescein (free acid) dye were acquired from Sigma-Aldrich for preparation of SRBs. The Harvard apparatus was obtained from Harvard Bioscience (Holliston, MA, USA), and silicone tubing (ID 1/32″) was purchased from Saint-Gobain Performance Plastics Laboratory Division (USA) for shaping the SRBs. Brachytherapy needles were purchased from IZI Medical Products (MD, USA) for the intra-tumoral administration of the SRBs. The lung (LLC1) mouse cancer cells (ATCC, USA) were cultured based on standard reported protocols (19). The monoclonal antibody anti-mouse CD40 (FGK4.5/FGK45) was bought from BioXcell (New-Hampshire, USA). All cell culture products (DMEM, Trypsin, Fetal Bovine Serum, penicillin/streptomycin, PBS pH 7.4) were obtained from Gibco, Thermo Fisher, and Life Technologies (Waltham, MA, USA).

### Fabrication of Smart-Multifunctional RT Biomaterials

Prototype SRBs were developed following previously reported procedures for loading drugs into RT biomaterials (15). The drug loaded was anti-CD40 mAb. SRBs with reservoirs were prepared with an assortment of different molecular weights of PLGA polymer with a blend of polar aprotic solvent systems. SRBs were fabricated by mixing 300 mg of PLGA with 3.5 mL of DMSO and 0.5 mL of chloroform to get a homogenous mix. The Harvard apparatus was used to reproducibly infuse prepared mixture at a constant flow rate into the silicon tubing with an internal diameter similar to that of currently used fiducials. The loaded silicon tubing was dried at 50°C for 48 h and then cut into lengths of 4 mm. The immunoadjuvant payload was loaded in the SRBs and both ends were sealed with excellent reproducibility. Details of different SRB designs have been reported in recent studies (2, 13).

### Cell Culture

C57BL background mouse Lewis lung carcinoma cell line, LLC1, purchased from ATCC were sustained in DMEM media supplemented with 10% FBS, 4 mM l-glutamine, and 1% penicillin and streptomycin solution (10,000 U/mL penicillin; 10,000 µg/mL streptomycin) respectively. Cells were grown in a humidified 37°C incubator under a 5% CO_2_ atmosphere.

### Animal Studies

Animal experiments were conducted in compliance with the guidelines and regulations set by Institutional Animal Care and Use Committee (IACUC). 8-week-old male C57BL/6NTac mice were purchased from Taconic Biosciences (Hudson, NY, USA) and were contained in a group of four in standard cages with free access to food and water and a 12 h light/dark cycle. All mice adjusted to the animal facility for at least 2 weeks before experimentation. All possible parameters that may cause social stress, like group size, among the experimental animals were carefully checked and evaded. Animals were observed three times a week after cell implantation for any physical abnormalities. Tumor models were generated by subcutaneously injecting LLC1 cells (5 × 10^4^ cells) into the dorso-lateral left and right flanks of each mouse. Tumor growth was regularly monitored until a tumor size of approximately 4 mm in diameter was reached. The mice were randomized and divided into four cohorts: no treatment, implant of empty SRBs, intra-tumoral injection of anti-CD40, and implant of SRBs loaded with the same concentration of anti-CD40. Another set of mice with similar cohorts had the right flank tumors irradiated at 5 Gy. All mice with immunoadjuvant treatment received 11 μg of Anti-CD40 mAb. The control groups each had four mice per group and the treated groups had five mice per group. In each inoculation, the anti-CD40 mAb was diluted in PBS to a final volume of 50 µl. The same volume of PBS was injected in the tumors of control and RT only groups. For mice treated with SRBs, the SRBs were implanted in the tumors using clinical brachytherapy needles.

### Tumor Volume Assessment

Prior to treatment and immediately after treatment, a digital Vernier caliper was used to measure the length and width of the tumor. The tumor was measured between the skin surface layers. The length was measured along the imaginary longitude of the leg; the width was measured in the direction of the latitude. The tumor volume was calculated using the formula: tumor volume = [1/2 * L *(W^2^)], where L and W are the length and width of the tumor, respectively. The tumor volume was plotted against time. Mice were monitored for tumor shrinkage and any skin condition that might appear after treatment. Tumor volumes greater than ca. 1 cm in diameter on each flank or tumor burst on either flank or necrotic tumors were determined as critical tumor burden and mouse was sacked following the protocol.

### Computed Tomography (CT) Imaging and Irradiation

A small animal radiation research platform (SARRP, Xtrahl, Inc., Suwanee, GA, USA) was used to irradiate four cohorts of the mice using 220 kVp, 13 mA, 10 × 10 collimator and 0.15 mm copper (Cu) filter at 5 Gy. The SARRP was also used to image all eight cohorts using 65 kVp and 0.8 mA. The CT imaging was conducted at every time point the tumor measurements were recorded. The CT images were analyzed using the Preclinical Imalytics software (20) to display the tumor volume comparing all the different cohorts of the study.

### Statistical Analysis

Statistical analyses for tumor volume were achieved using standard Student’s two-tailed *t*-test. *P < 0.05, **P < 0.01, ***P < 0.001 was considered as statistically significant. Survival data were plotted and statistical analyses were performed using GraphPad prism v7.0. The Kaplan–Meier statistics (Madsen 1986, Statistical concepts Prentice Hall, Englewood Cliffs, NJ, USA) was utilized. A log-rank test was employed to determine the *p*-value for the Kaplan–Meier curves.

## Results

**Figure 1:**
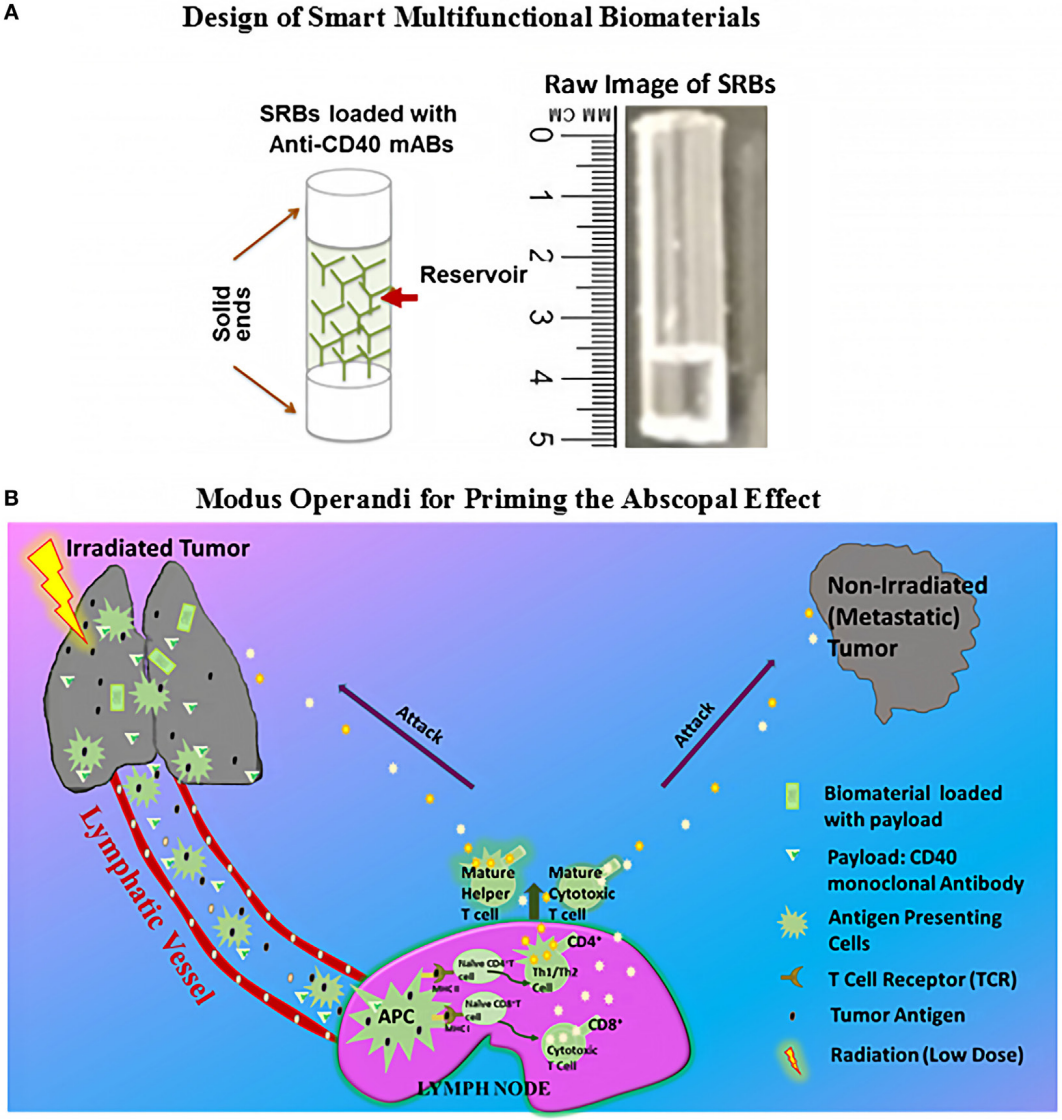
Design of multifunctional smart radiotherapy biomaterials and *Modus operandi* of treatment. **(A)** Schematic of smart radiotherapy biomaterials biomaterial (SRB) loaded with CD40 mAbs, and on the right, is the raw image of the empty SRB. **(B)** *Modus operandi* for using loaded SRB_CD40 mAbs and image-guidance radiotherapy (RT) to treat the primary tumor. RT generates antigens which can be taken up by antigen-presenting cells (APCs). The CD40 mAbs support APC maturation followed by cross presentation to T cells in the lymph node. Mature T cells can then kill cancer cells both locally and at distant sites.

Figure 1A shows a schematic and prototype SRB designed for use in this study with a capacity for the high loading volume of payloads sufficient to prime significant abscopal responses. Figure 1B illustrates possible *Modus operandi* for enhancing the abscopal effect using SRB (3, 21). Neoantigens and danger signals help to elicit antigen-presenting cells (APCs) are generated by RT (8, 22) as illustrated in Figure 1B. The antigens can be engulfed by the APCs, followed by the presentation of the antigens through the major histocompatibility complexes (MHC I/MHC II) to CD4 T or CD8 T cells in the lymph node (12).

**Figure 2:**
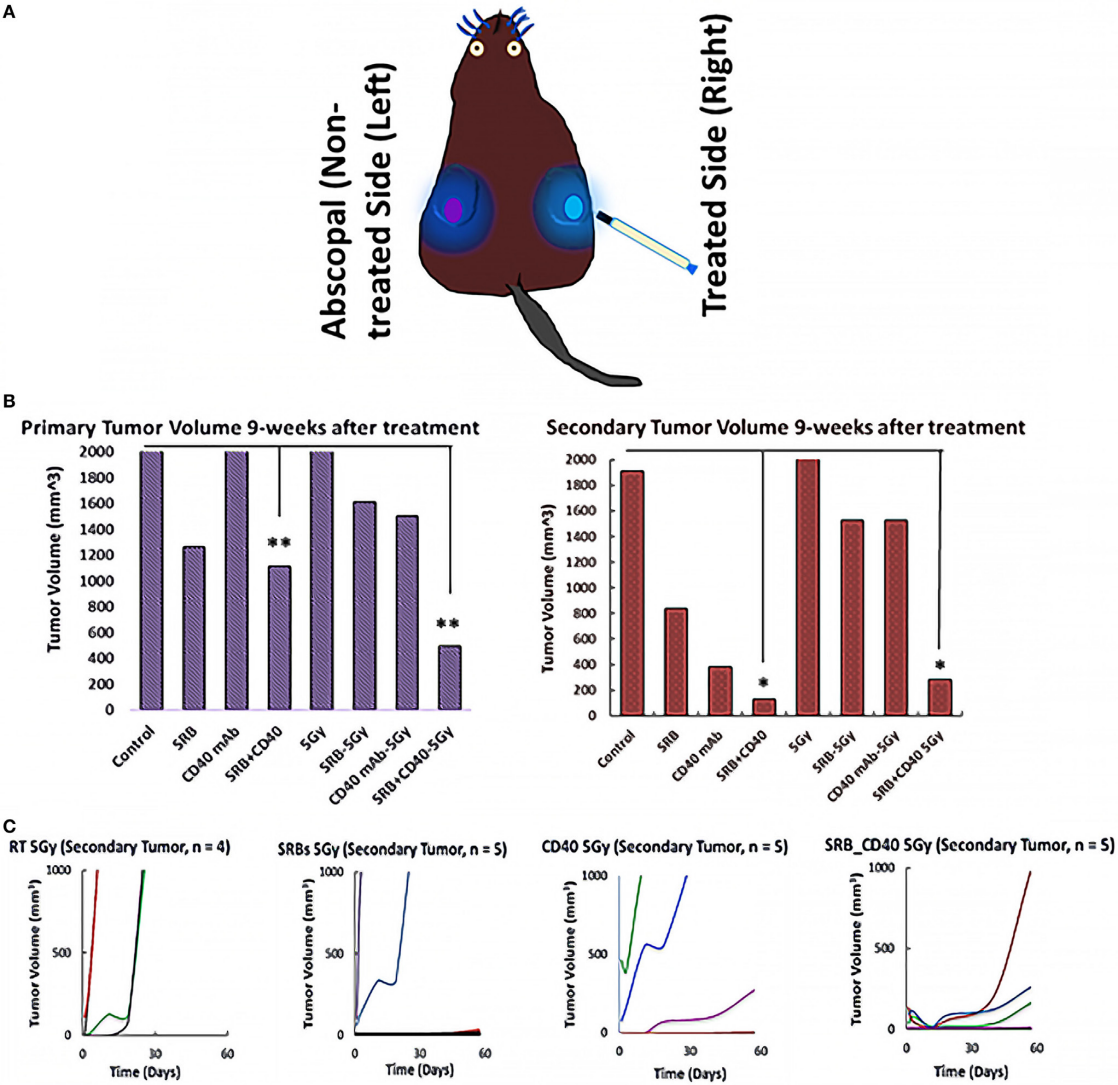
Tumor volume responses 9 weeks after treatment. **(A)** Schematic depiction that represents the treatment (blue) and abscopal site (purple). **(B)** Represents average tumor volume of each cohort monitored 9 weeks after the start of treatment of either anti-CD40/smart RT biomaterials/RT or their combinations thereof. **(C)** Denotes the secondary/metastatic tumor volume (non-treated tumor) for each mouse in the irradiated cohorts at 5 Gy 9 weeks post treatment.

Figure 2A shows a cartoon of a mouse with bilateral tumors, where the right flank tumor is treated and the left flank tumor is not treated as described above. Figure 2B shows the average tumor volume for each cohort 9 weeks after the start of treatment. Tumor volume post 9 weeks treatment represents tumor volume measured before the mouse died or was euthanized. An increased abscopal effect is observed (Figures 2B,C) in cohorts treated with SRB loaded with CD40 mAb. The results suggest that the use of SRBs loaded with CD40 mAb can boost the abscopal effect.

**Figure 3:**
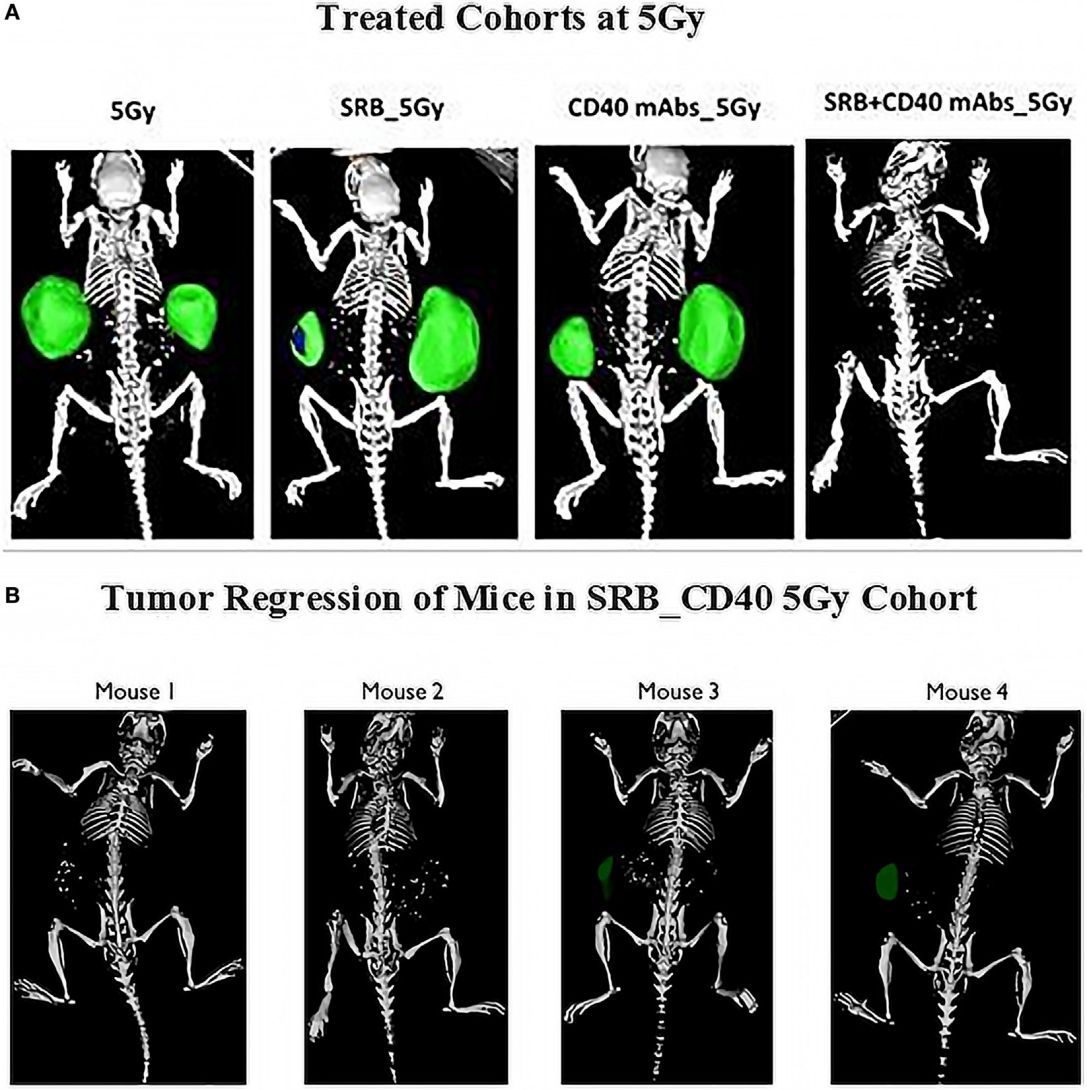
Computed tomography (CT) images of mice at 9 weeks after treatment. **(A)** Whole body CT images depicting: 9 weeks after treatment with (SRB; CD40; SRB_CD40) at 5 Gy. **(B)** Four out of five mice in the SRB_CD40 mAb group treated with image-guided radiotherapy. One mouse is excluded due to high tumor burden before the start of treatment.

Given the interest in specifically investigating the abscopal effect during RT, the processed CT images (Figure 3) illustrate differences in tumor regression for animals treated with SRBs during RT compared to other cohorts treated with RT, but without SRBs. The most significant difference was observed for the cohort of mice irradiated with SRBs loaded with CD40 mAb (Figure 3A). Meanwhile, Figure 3B shows the processed CT images of four mice belonging to this group, where RT of 5 Gy was used along with SRBs loaded with CD40 mAb (the SRB_CD40_5 Gy cohort). One mouse (not shown) out of five did not respond to treatment.

**Figure 4:**
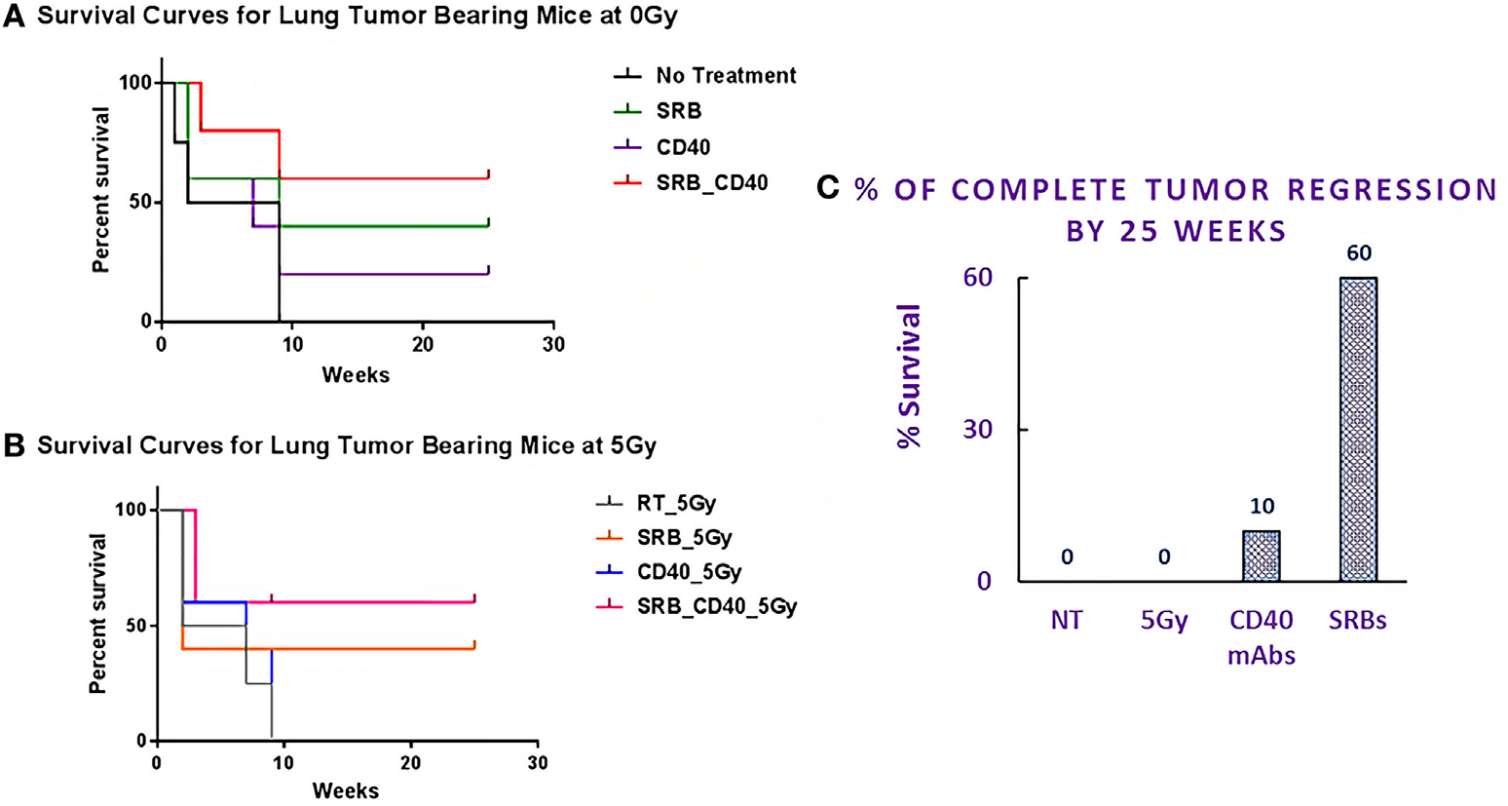
Mice survival curves. Survival curves for mice in **(A)** all four cohorts with no radiotherapy (RT), **(B)** comparing another four similar cohorts exposed to 5 Gy dose. **(C)** Plot of percentage of mice that showed complete tumor regression during 25 weeks observation period. Henceforth, during the observation period of 25 weeks, 60% of the mice that showed complete tumor regression came from all the SRB cohorts with or with RT or CD40 mAbs or their combinations thereof. However, 10% of mice, 1 mouse out of 10 treated with CD40 mAbs (with or without RT) showed complete tumor regression. The control and the RT cohorts showed no tumor regression during the observation period. The correlation that was observed for these mice showing complete tumor regression was due to smaller sized tumors (2–6 mm) at the point of treatment. At higher tumor size, the response was not as favorable as observed in this study.

Survival results for different cohorts are shown in Figure 4. Even without using RT, mice treated with SRBs showed increased survival compared to mice treated with CD40 mAb without SRBs (Figure 4A). Possible explanation for the observed response to SRBs without radiation is discussed below. Meanwhile, Figure 4B shows clear advantage in the use of SRBs during RT compared to other cohorts treated with RT alone or RT and CD40 mAb without SRBs. The results showed increased benefits when using SRBs with CD40 mAb during RT. Altogether, these results reinforce the tumor volume data indicating that the use of SRBs is beneficial in increasing survival (22, 23). The highest percentage of survival 25 weeks post-treatment was observed in mice treated with SRBs (Figure 4A). After 25 weeks, 60% of the mice from the SRB cohorts showed complete regression compared to 10% for cohorts administered with anti-CD40 mAbs, but no SRB (Figure 4C). Complete tumor regression was not observed in any other cohorts (Figure 4C).

## Discussion

The results of this study provide the first experimental evidence demonstrating the potential of SRBs loaded with anti-CD40 to boost abscopal responses. The advantages in the use of SRBs versus intra-tumoral administration have been highlighted in recent studies (3, 4). One advantage sustained release of CD40 mAb from SRB which enables the persistent and the contemporaneous presence of antigen and adjuvant signaling in the tumor microenvironment, during RT (5, 6, 24). The sustained delivery advantage is predicated on promising results from vaccine studies, which have shown that sustained delivery of a vaccine using biomaterials elicits increased proliferation of antigen-specific CD4^+^/CD8^+^ T cells compared to direct systemic injections (18, 22). Besides this, the polymeric biomaterial component of SRBs may also support the maturation of dendritic cells (DCs) when the DCs are exposed to such polymers (23). The maturation of such DCs like the APCs would prove favorable in priming the abscopal effect (12, 21, 23, 25).

One interesting observation in this preliminary study was the fact that even without RT, SRBs with CD40 mAbs, could engender an immune response with survival results comparable to those of mice in the cohort treated with RT and SRBs loaded with CD40 mAbs. This may be because, implantation of the SRBs can cause inflammation or render cells to release cellular danger-associated molecular patterns (DAMPs) and cytokines that enhance traffic of immune cells (26). In the groups treated with SRBs and anti-CD40, the inflammation or DAMPs may contribute to the immune response observed, minimizing the additional benefit of RT. The advantage of using RT is that it is expected to generate more neoantigens, broadening the repertoire of responding T cells, and enhancing efficacy. The use of other doses of RT may provide a clearer additional benefit. However, this possible explanation warrants further investigation, e.g., in investigating the immune-cell populations engaged by the SRBs alone compared to those engaged when the SRBs are not present. The findings also underscore the need for further studies to further elucidate the biological mechanism underlying the abscopal effect. The only clear finding here is that the use of the specific SRBs used in this study may promote a beneficial immune response.

As highlighted in our recent work (2), using immunotherapy to boost the abscopal effect, multiple preclinical studies have investigated different dose and fractionation regimens in different cancer types. Generally, larger doses per fraction were associated with abscopal effects. However, abscopal effects have also been observed for lower single doses of RT, e.g., at 4 (27) and 6 Gy in our previous study (18) with no SRB. In this current study, we decided to use another single dose close to 6 Gy with SRBs. As the results show, an abscopal effect was also achieved. However, it is possible that other RT doses could further enhance the treatment outcomes. For each tumor type, an optimal dose range is likely to exist, below which immune stimulation might be suboptimal and above which immunosuppression prevails. Further investigations of dosage and combinations of RT with immunoadjuvants are needed to determine the optimal thresholds or range (2, 28).

A limitation of this preliminary study is the smaller number of animals used in each cohort. The control and the RT groups only had four mice in each cohort. The other groups had five mice per cohort. Another limitation is the lack of immune-cell population analysis taken for each cohort. Ongoing studies are targeting cohorts with larger number of mice with such analysis planned. Other factors that may influence the priming of a robust abscopal response include: the tumor burden at the start of treatment, the doses, and scheduling. More studies are needed to optimize these parameters for priming the abscopal effect. The use of SRBs may obviate the need for repeated injections or timing considerations for the administration of the immunoadjuvants in cancer treatment.

If the approach to use SRBs loaded with CD40 mAb to boost the abscopal effect is translated clinically, this could transform RT practice, extending the use of RT to the treatment of both local and metastatic tumors. The clinical impact would be significant for many more cancer patients, since metastasis accounts for >90% of all cancer-associated deaths (25). Our ongoing studies with larger cohorts may lead to improved abscopal response rates, allowing the minimization of systemic/overlapping toxicities using this approach. Most importantly, this approach using SRBs could be employed in treatment of pancreatic, breast, prostate, and liver cancers, where inert RT biomaterials are currently employed.

The results of this study provide evidence that the use of SRBs loaded with immunoadjuvants like anti-CD40 mAbs could enhance both local and metastatic tumor cell kill. Such SRBs could be developed to replace currently used inert RT biomaterials, e.g., fiducials, spacers, and beacons, at no additional inconvenience to cancer patients. RT is mainly used for the treatment of localized disease. The use of SRB approach demonstrates the potential for extending RT to the treatment of metastatic disease.

## Ethics Statement

This study was carried out in accordance with the recommendations of Dana–Farber Cancer Institute IACUC. The protocol was approved by the Dana–Farber Cancer Institute IACUC.

## Author Contributions

SY-K provided intellectual contributions to the design of the mice study and reviewed the manuscript. SK analyzed some of the CT images and reviewed the manuscript. NS analyzed some of the CT images. RK reviewed the manuscript. FG designed and invented the Imalytics Software used to analyze the CT images. KC reviewed the manuscript. WN is the principal investigator who designed the study and reviewed the manuscript. MM generated all the results in this study, designed the multifunctional RT biomaterial (SRB) to implant in mice, and wrote the manuscript.

## Conflict of Interest Statement

The authors declare that the research was conducted in the absence of any commercial or financial relationships that could be construed as a potential conflict of interest.
